# Survive or die? c-MYC has the last word

**DOI:** 10.1038/cddis.2015.366

**Published:** 2015-12-17

**Authors:** S Wu, X Yin, X Liu, L Chu

**Affiliations:** 1 State Key Laboratory of Cell Biology, Institute of Biochemistry and Cell Biology, Shanghai Institutes for Biological Sciences, Chinese Academy of Sciences, Shanghai, China; 2 Jiangsu Center for the Collaboration and Innovation of Cancer Biotherapy, Cancer Institute, Xuzhou Medical College, Xuzhou, China

Metabolic alteration is one of the important characters in cancer cells, which confers advantages for survival and proliferation of cancer cells. Altered metabolism is believed to support the bioenergetic and biosynthetic demands of rapid proliferation of cancer cells. Aerobic glycolysis provides a large amount of intermediates for the synthesis of nucleic acid, amino acid and lipid that are required for cell replication.^1^ Aerobic glycolysis is also a good way to adapt the hypoxic condition.^2^

A lot of work demonstrated that metabolic change might be an adaption to tumor microenvironment and aberrant oncogene activation or tumor suppressor loss.^3^ Most metabolic enzymes and regulators are the targets of oncogenes or tumor suppressor genes.^4^ For example, oncogene c-MYC, which is deregulated in most of human cancers, was reported to drive the cancer cell metabolic reprogramming.^5^ However, more and more data show that the interaction between metabolic alteration and tumor microenvironment or oncogene/tumor suppressor is mutual. Metabolism is not only regulated by tumor microenvironment or oncogenic pathways, but also an upstream regulator that affects the cellular activity. c-MYC was previously demonstrated to promote both glucose metabolism and glutamine metabolism.^5^ Recently, a *Cell Death Discovery* article by us showed that c-MYC was differentially affected by glucose deprivation (GD).^6^ An interesting finding of our work is that GD decreases c-MYC protein levels in some cancer cell lines, but increases c-MYC in other cancer cell lines (Figure 1). We further found that GD differentially affected c-MYC protein stability through affecting c-MYC phosphorylation in different cancer cell lines. By chemical molecules screen, we discovered that PI3K and SIRT1 can affect c-MYC phosphorylation and inhibit its degradation through proteasome. In addition, the different change of c-MYC might account for the cell sensitivity to GD.

Glucose and glutamine are two main nutrients to support cell growth and division.^7^ If glucose is depleted, cells will use glutamine to meet their demands. Glutamine is not only a carbon source, but also a nitrogen donor.^8^ c-MYC regulates glutamine reprogramming by directly stimulating the glutamine transporter genes and indirectly inducing glutaminase 1 (GLS1).^5^ We discovered that c-MYC was induced by GD in MDA-MB-231 cells. This might promote cells to use glutamine metabolism to fuel tricarboxylic acid (TCA) cycle. Yuneva *et al.* showed that addition of TCA cycle intermediates can rescue glutamine-depleted cells from apoptosis.^9^ GD-induced c-MYC upregulation might be a strategy for cancer cells to convert glucose metabolism into glutamine metabolism and survive when glucose is limited. However, c-MYC protein levels were decreased in response to GD in HeLa cells. This made HeLa cells unable to use glutamine to replenish TCA cycle and then cells were prone to apoptosis under GD condition. We concluded that the response of c-MYC determined the sensitivity of different cancer cell lines to GD.

c-MYC protein level is mainly regulated by post-translational modifications, especially phosphorylation and acetylation. Several molecules are reported to regulate the modification of c-MYC, including GSK3,^10^ ERK^11^ and SIRT1.^12^ We found that GD decreased c-MYC phosphorylation and protein stability in HeLa cells and these effects can be inhibited by PI3K inhibitor and SIRT1 inhibitor. However, c-MYC protein stability was not affected in MDA-MB-231 cells. Our work left two open questions. First, why different cells show opposite response to GD on c-MYC protein level? We provided evidence that c-MYC protein stability was differentially affected in different cells. We analyzed several cancer cell lines and found that the different response of c-MYC under GD is independent of P53, KRAS and PIK3CA. However, so far, we were not able to figure out the detailed heterogeneity of different cells that determined the response of c-MYC to GD. Second, it still needs to further investigate that whether change of c-MYC in response to GD can be used as an indicator of the sensitivity to GD in different cells. The dependency of different kinds of nutrition varies with cell lines. For example, some cells are sensitive to glucose depletion, while other cells are sensitive to glutamine depletion. The heterogeneity of different cells makes it difficult to get a consistent conclusion obtained from a few cell lines.

We might study the metabolic heterogeneity of cancer cells in an evolutionary way. It is not clear that how cancer cells switch the metabolic pathways from oxidative phosphorylation to aerobic glycolysis. Different cancer cells use different metabolic pathways and show distinct sensitivity to nutrition stress. However, few studies focus on the transition of both metabolic pathways in one system. In addition, how do cancer cells switch glucose addiction to glutamine addiction? The difficulty is that the transition of different metabolic pathways might not be dependent on single factor.

Targeting cancer metabolism is a promising approach for cancer treatment. Understanding the molecular mechanism of metabolic regulation in cancer cells will help us find more effective therapies. Our work suggested that the change of c-MYC might be thought as an indicator of choosing targeting metabolism for cancer therapy. For cancer cells, such as MDA-MB-231 cells, a combination of targeting glucose metabolism and glutamine metabolism might be an effective way for cancer therapy.

## Figures and Tables

**Figure 1:**
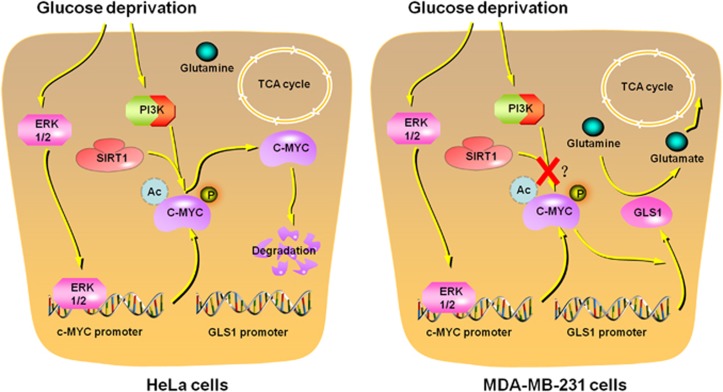
Glucose deprivation (GD) affects c-MYC protein levels in a cell-type-dependent manner. GD dephosphorylates and then decreases c-MYC protein stability through PI3K signaling pathway and SIRT1 in HeLa cells. In MDA-MB-231 cells, c-MYC protein is protected by undetermined factors and is not affected by PI3K and SIRT1. Accumulation of c-MYC promotes glutamine metabolism through indirectly inducing glutaminase 1 (GLS1)
